# Sustainable transformation of agriculture requires landscape experiments

**DOI:** 10.1016/j.heliyon.2023.e21215

**Published:** 2023-10-24

**Authors:** Anna Pereponova, Kathrin Grahmann, Gunnar Lischeid, Sonoko Dorothea Bellingrath-Kimura, Frank A. Ewert

**Affiliations:** a Leibniz Centre for Agricultural Landscape Research (ZALF), Eberswalder Str. 84, 15374, Müncheberg, Germany; b University of Potsdam, Institute of Environmental Science and Geography. Campus Golm, Karl-Liebknecht-Str. 24-25, 14476, Potsdam, Germany; c Humboldt University of Berlin, Department of Agronomy and Crop Science. Albrecht Daniel Thaer-Institute of Agricultural and Horticultural Sciences, Invalidenstraße 42, 10115, Berlin, Germany; d University of Bonn, Institute of Crop Science and Resource Conservation (INRES), Karlrobert-Kreiten-Strasse 13, 53115, Bonn, Germany

**Keywords:** Ecosystem services, Sustainable intensification, Knowledge generation, Agricultural research, Landscape scale

## Abstract

Transformation of agriculture to realise sustainable site-specific management requires comprehensive scientific support based on field experiments to capture the complex agroecological process, incite new policies and integrate them into farmers’ decisions. However, current experimental approaches are limited in addressing the wide spectrum of sustainable agroecosystem and landscape characteristics and in supplying stakeholders with suitable solutions and measures. This review identifies major constraints in current field experimentation, such as a lack of consideration of multiple processes and scales and a limited ability to address interactions between them. It emphasizes the urgent need to establish a new category of landscape experimentation that empowers agricultural research on sustainable agricultural systems, aiming at elucidating interactions among various landscape structures and functions, encompassing both natural and anthropogenic features. It extensively discusses the key characteristics of landscape experiments and major opportunities to include them in the agricultural research agenda. In particular, simultaneously considering multiple factors, and thus processes at different scales and possible synergies or antagonisms among them would boost our understanding of heterogeneous agricultural landscapes. We also highlight that though various studies identified promising approaches with respect to experimental design and data analysis, further developments are still required to build a fully functional and integrated framework for landscape experimentation in agricultural settings.

## Graphical abstract

**Figure:**
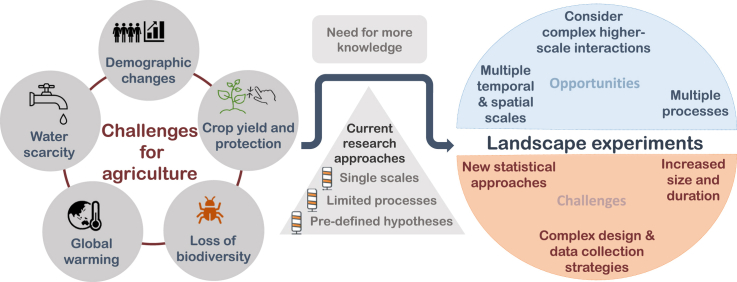

## 1 Introduction

Sustainable agriculture seeks to meet the growing demand for food, feed and fibre of future generations while minimizing both environmental impacts and economic risks for farmers. To achieve this, sustainable agricultural systems should be resource-efficient, resilient, and productive, as measured within their social, environmental and economic dimensions [1,2]. In practice, transformation to sustainability requires the adoption of management frameworks, which would allow balancing between objectives of each dimension and consider (unexpected) interactions between them. So far, existing agricultural policy has not been able to solve the problems caused by agricultural intensification and to adapt farm-level management decisions that support sustainable practices [[3], [4], [5], [6]].

Achieving sustainable agriculture represents a considerable challenge for scientists and policy-makers, while also requiring an active involvement of farmers [7,8]. While many studies tried to analyse these dimensions simultaneously, they often reported differing conclusions due to the use of limited proxies [2] or other issues (please see Chapter 3 - Shortcomings of current research and experimental approaches). Providing sufficient scientific evidence is crucial to advance existing practices and policies. It will require scientists to develop or adopt new modes of research to interact more closely with farmers and policymakers which should aim to be knowledge- and change-oriented or intermediating [9].

Specifically, to avoid gaps in innovation processes, agricultural experimentation needs to consider the dependence on local nature and environmental contexts, which creates many obstacles in real-life implementation. For example, there is often a mismatch between the spatial scale of ecological processes and that of agricultural management in experimental design and data collection [10]. Ecological processes such as water and nutrient transport or landscape structures relevant for biodiversity span a wide range of spatial scales, while agricultural management acts at the field and farm scale (see e.g. Ref. [11]). On the other hand, policies are developed and implemented at the scale of administrative regions. Thus, new management approaches to facilitate the redesign of agricultural production systems need to be tested at a variety of spatial and organisational scales [4,[12], [13], [14]] to close the gap between field/farm and region. Moreover, diverse sources of knowledge and methods need to be combined to re-design cropping systems towards more sustainable management (see e.g. Ref. [15]).

This study aims to examine existing methods, challenges and potential opportunities for experimentation in agricultural landscapes to support the transformation of agriculture. The goal is to support researchers in identifying avenues to set up meaningful experiments that can enhance our understanding of interacting processes in agroecosystems, support policy decisions, and improve recommendations for farming practices with limited trade-offs for sustainable agriculture. To achieve these objectives, a critical literature review [16] was conducted, and the following research questions are addressed:

1. What are the major research needs to support the development of sustainable agricultural practices?
2. What are the major shortcomings of current approaches to satisfy these research needs?
3. How can current shortcomings in regard to experimental design, data collection and analysis be overcome?

We describe landscape experiments as a promising approach to address these shortcomings on the way to sustainable agriculture in a systemic way.

## 2 Current state and the need for broadening the knowledge base

Among existing concepts, aiming to achieve different goals of sustainable agriculture (e.g., organic agriculture [17] or sustainable and ecological intensification [[18], [19], [20], [21]]), agroecology is widely recognised as an all-encompassing approach, which considers “how agricultural practice and research can be geared to the needs of people and the planet” [22]. It links nature and technology, productivity and natural resources, scientific and traditional knowledge, social and cultural values [[22], [23], [24], [25]]. Though agroecology encompasses the global food system at its highest level, its lower levels describe the practical steps that farmers can take within their farms [26], at the scales of field or farm and beyond. Addressing the higher-level social changes requires a thorough understanding of the means of how to operate these changes at the lower levels, which constitutes the science and practical aspects of agroecology [24,27]. The first key step for this is a better understanding of the underlying relationships between the biotic and abiotic elements of agroecosystems in a systematic way and testing and development of agricultural practices that both satisfy the food demand and are environmentally and economically sustainable [24].

However, existing studies on agricultural systems, mainly relying on experimentation in generating new knowledge, do not yet fully address the complexity of real working agroecosystems. While classical small-scale laboratory-to-field trials are mainly used to address agronomic questions, larger scale experiments focus rather on environmental issues (a detailed classification of experiments is provided in Chapter 4.1). Recent literature suggests that agroecosystems require more systematic thinking about natural and human-related factors and processes at various scales in their interaction, which is often associated with the landscape scale [8,11]. The understanding of landscape, however, varies among the different disciplines included under the umbrella of agroecology, and even depending on the specific research topic. For the approaches discussed in this paper, *landscape* is defined as *a system of spatially arranged entities which are structurally and functionally interconnected* (Pereponova et al., 2023, in review). Thus, it determines the system boundaries by considering important interrelations between the processes of different scales, rather than assigning a strict spatial definition to it. This way of defining landscape acknowledges the complex and dynamic organization of agroecosystems, which needs to be accounted for while conducting research in the context of multiple goals of agroecology.

To compensate for the lack of scientific knowledge on sustainable agroecological practices further empirical data from experimentation is required [28,29], which should satisfy certain requirements. This data should reflect the diversity of processes (energy flows, nutrient cycling, population-regulating mechanisms, system resilience), providing evidence for trade-offs and synergies between the relevant agroecosystem components. To capture the synergies and trade-offs, it is necessary to integrate knowledge from different disciplines in a systematic way, while addressing conflicting methodological standards. New practices and technologies should account for potential longer-term effects (such as climate change) and be tested in real-life environments and adapted or fitted to local conditions [4,14]. However, current experimental approaches do not meet these requirements in an adequate way and largely mismatch with the relevant spatial and temporal domains of relevant processes in agricultural landscapes.

## 3 Shortcomings of current research and experimental approaches

This chapter provides an overview of specific problems of the current experimental studies. Agricultural field experiments have limited applicability to answer the questions related to our transition towards sustainable landscapes. Classical small-plot field experiments and conventional statistical approaches, for example, cannot capture spatial and temporal variability of larger scale processes occurring in farming and landscape systems (the classification of experiments in agriculture is provided in subchapter 4.1 - Differentiation of landscape experiments from “classical” field experiments). Also, treatment effects in such experiments may be sensitive to scale or boundaries, which makes the results unreliable or even risky for end users [30].

Most often, agricultural field experiments focus on individual fields and the associated land only and consider different fields or patches as functioning in isolation and ignore their connection via spill-over effects (e.g., chemical drift, prey, pollinators, wildlife, seedbank) [31]. Plot experiments may be misleading in regard to evaluating multiple ecosystem services due to the high degree of variability of natural conditions within and between landscapes [32]. Often, different aspects are investigated separately, such as ecological goals (e.g., biodiversity) and agricultural production targets (e.g., yield) of land use. Alternatively, the analysis of relationships between them is limited to two or three factors simultaneously included in a study [33]. Additionally, though the need to address non-linearity of interactions between such processes has received recognition, linear techniques are still used in many cases for analysis of the results, which may lead to erroneous assumptions [34]. This is a major obstacle to developing multi-functional landscapes which contribute to the evolvement of both, productive and sustainable agricultural systems.

Below we review in more detail some apparent shortcomings of existing experimentation approaches related to the diversity of scales, cause-and-effect relationships, and suitability of indicators and models.

### 3.1 Scale

Existing studies on agricultural landscapes mainly focus on single sites chosen based on the assumed relevant spatial scale of particular processes and ignore long-term effects concerning the diversity of processes differing in temporal scales [35,36]. Thus, cause and effect areas are often not allocated precisely, the empirical data is spatially dispersed, and a systematic evaluation is lacking [36]. Agronomic field studies considering smaller spatial and temporal scales may lead to erroneous conclusions, depending on the selected metrics (see e.g., Ref. [37]).

**Fig. 1:**
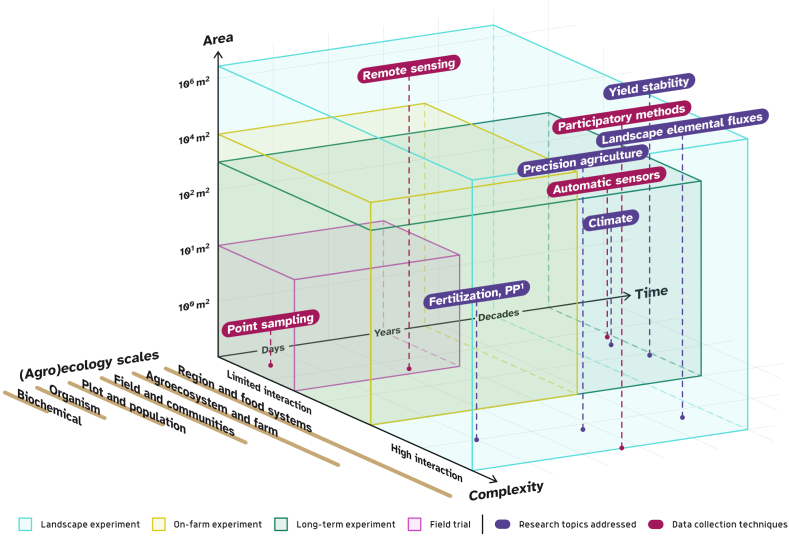
Selected research topics, addressed by agronomic and agroecological studies, and data collection techniques within temporal, spatial and complexity scales. Time and Area axes reflect temporal and spatial scales respectively; Complexity axis shows to which degree interactions between various entities are considered: from a limited (1–3) number of effects considered at a time to multiple agricultural and environmental parameters, defining agroecosystem functioning; Next to the complexity axis are ecology (agroecology) scales, respectively: biochemical; organism; population (plot); communities (field); ecosystems (farm, agroecosystem); food system and region); *PP refers to plant protection. Different types of experiments may be used in a combination, thus, e.g., field trials may be included within LE.

Meanwhile, for the complex topic of agroecology, choosing the right spatial and temporal scale to study is crucial, as processes at different spatial, temporal and complexity scales are involved [11,38]. Here we define scale following [39] as the “spatial, temporal, quantitative, or analytical dimensions used to measure and study any phenomenon”, while “units of analysis that are located at the same position on a scale” represent levels [39]. Depending on a specific question, organisational levels of ecological, and agroecological processes span from the micro-level to the field (controlled mainly by biophysical relationships), farm (management decisions level), landscape, region, or continental and global levels [[39], [40], [41]]. The levels affect each other and involve processes of growing (often simultaneously) spatial, temporal and complexity scales. The latter here is determined through the number of interactions between the entities defining the system at a given level. Ecological complexity scales span from the metabolism of single microorganisms to ecosystems [42], and typically grow with increasing spatial and temporal scales Agricultural context adds additional complexity of management and social perspective, from the level of cultivation practices (with basic consideration of physical and biological factors) to the one of agri-food system (with inclusion of different actors) [27] (Fig. 1).

In respect to spatial scales in agricultural research, small plots usually serve to test separate agronomic practices and the farm is often used as a research entity to consider management decisions from different perspectives (e.g., Ref. [43]), e.g., for the evaluation of nitrogen dynamics and balances [44,45]. Such approaches, however, miss spatio-temporal changes and further environmental consequences at a larger scale [41].

In regard to temporal scales, agroecosystem processes span from fast small-scale (preferential solute transport in the soil, microbial processes) to meso-scale biophysical processes (fire, storm, insect outbreak) and those taking years to centuries (soil carbon sequestration, soil formation) within landscapes and regions, and can be cyclic or continuous [28,35,46,47]. Since some changes may bring short-term disadvantages, but long-term benefits, regular and continuous tracking is needed [48].

As there is no single natural scale to study natural systems, scaling of measurements is often used [38,49]. Extrapolation of point measurements is applied when data are prohibitively time-consuming and expensive to collect and limited in spatial and/or temporal scale [50]. Simple linear extrapolation of point measurements is, however, prone to errors [37,51], and becomes unreliable under heterogeneous conditions, especially in case of nonlinear dynamics and major changes of constraints [49]. Sometimes changes at a specific scale may cause a system to suddenly reorganize around alternative mutually reinforcing processes (see e.g., Ref. [46]).

Investigating responses to temporal changes requires a series of inventories over time. Alternatively, inference about the treatment effect can be done through space-for-time substitution [47,52], using a ‘snapshot’ at a single time to compare sites at different stages. This, however, may not cover the diversity of defining changing conditions, such as the manifold effects caused by climate change and the uncertainty surrounding predictions thereof. The very rate of environmental changes may also lead to shifts in system states (“rate-tipping”) across scales [53].

To sum up, the choice of experimentation scale is critical to get reliable and meaningful results. If based solely on the scale of the specific process under study, it may lead to erroneous conclusions. It is, thus, crucial to identify the key related processes and the smallest common spatial and temporal scale to build the experimental design upon. Often, the landscape or region, determined both by natural conditions and land use [36], is proposed as the optimal scale for systemic research of complex topics (e.g. Refs. [35,54]).

### 3.2 Mono-causality

Our knowledge about environmental dynamics and processes builds on decades of extensive research. In natural systems, numerous processes interact at different scales in a complex way. Consequently, even after decades of research, our knowledge is still far from sufficient to develop sustainable management schemes. For illustration, please see Ref. [54] in terms of nitrous oxide emissions from soils. Another example, by Ref. [55] highlighted the still incomplete exploration of the role of biological interaction systems in soil-related ecosystem services, with a focus on invertebrates. This lack of knowledge may lead to controversial effects of management strategies “focused only on the symptoms”. These interactions become particularly complicated in the case of agricultural systems, where natural ecosystem complexity is increased by a cultural component, such as agricultural management (e.g., Ref. [56]).

Current empirical studies usually consider only a very small number of drivers and response variables each. For example, Weibull et al. [57] mention that the majority of studies on species richness in agroecosystems focus either on the effect of farming systems or landscape features, while in fact, a particular combination of these factors defines the relative importance of their effects. Rillig et al. [58] found that out of more than 4000 studies on the effects of stressors on soil biota and processes published between 1957 and 2017, only 20 % considered more than one single stressor, and less than 2 % considered three or more factors and interactions between them. Meanwhile, they found in their own experiments that single effects did not only simply add to each other but in many cases reinforced harmful effects of other stressors in a non-linear way. Similarly, Liess et al. [59] explain the higher-than-expected sensitivity of vulnerable stream species to pesticide effects through non-additive, but synergetic toxicity effects of multiple pesticides, as well as of environmental stressors. More generally, Bennet et al. [34] demonstrated that most studies on ecosystem services either ignore the significance of relationships between them or limit the analyses to one or two, often using land use/cover as a proxy and assuming linear relationships with ecosystem structure. The authors classified possible relationships as 1) based on common drivers or 2) on interactions among ecosystem services, positive or negative, mono- or bi-directional. Even when such relationships are assumed in studies, the mechanisms behind them are typically not assessed. This is, however, critical to avoid unexpected dramatic changes. Lischeid et al. [60] used a machine learning approach to check the predictive power of joint consideration of single effects proven to have significant effects on small lake water quality in preceding studies. For none of the nutrients or major solutes, the model succeeded in explaining more than 6 % of the variance. Synodinos et al. [53] described the role of tipping mechanisms, where the nature of change in one factor may lead to catastrophic changes of the system. These examples highlight the limited transferability of single-process studies into high-dimensional real-world settings.

To conclude, the need for understanding and managing ecosystem services focusing on manifold relationships rather than separate elements, and built upon integrated system approaches to support ecosystem resilience and efficiency, is a theme that starts to pervade scientific thinking in respect to agricultural management (e.g., Refs. [34,61,62]). However, it is not yet well addressed by research in practice.

### 3.3 Indicators

The complexity of environmental systems and their interactions with anthropogenic activities is a major challenge not only for science but for management and policy in terms of defining goals and assessing the efficacy of management schemes and policies. For that purpose, indicators have widely been introduced. Indicators are relatively easy to measure properties of complex systems that are closely related to complex system's functions, e.g., biodiversity or soil health in general, which are not directly accessible at reasonable expense [63,64]. Examples for the case of ecosystem services are provided in Refs. [36,65], or for soil quality in Ref. [55].

To be practically useful, indicators should satisfy certain requirements which in practice are not always met.

Particular caution is recommended to balance ease of measurement and assessment with representativity. Indicators should be applicable across a wide range of conditions, yielding comparable results [66]. The applicability and usefulness of indicators are also not entirely independent from the local context. The basis for the current agri-environmental indicator framework at EU scale is provided by OECD's DSR (Driving force-State-Response) and the European Environment Agency's DPSIR (Driving force-Pressure-State-Impact-Response) framework. It aims to capture the main factors that define the relationships between agriculture and environment [48], however, the discussions about particular indicators and their ease of assessment and representativity are still ongoing [67]. The Human Development Index is a paramount example for a long-lasting discussion about shortcomings and necessary modifications of a widely used indicator as well as for the development of a plethora of alternative indicators [[68], [69], [70]].

With respect to agroecology, Wiget et al. [71] listed such “key aspects'' of indicator-based assessment frameworks as adaptability to local conditions, farmers’ involvement, consideration of multiple functions of agroecosystem and analysis of interactions among the multiple functions and their respective indicators, which are not satisfied by the majority of existing frameworks.

An integrated approach (as described in the previous subchapter) to the evaluation of the transition to sustainable agriculture requires the involvement of different disciplines and related indicators. Indicators, covering a selected sustainability aspect, often do not provide a complete estimation, e.g., some negative effects are externalized and not considered [48]. Scale considerations are also important for defining the minimum set of indicators to describe the overall landscape status [72,73] and different ecosystem services without being redundant. Already while working at the agroecosystem level, the social dimension of assessment should not be ignored, to allow further harmonisation of integrated assessment frameworks. While social dimension and relevant indicators are currently getting significant attention from researchers, often a lack of bridging to other scales and relevant indicators can be observed [74,75], though this issue has been addressed in some works (see e.g., Ref. [74]).

Given the dynamic character of agroecosystems, indicators should reflect the current state, but also trends concerning environmental, management-related, socio-economic or policy changes [71,72]. Additionally, the ability of an indicator to provide early warning signals is important to timely inform about possible undesirable changes [76]. Besides, the very rate of changes, which may also lead to shifts in the system as a whole (see e.g., Ref. [53]), is seldom considered [77].

Another practical requirement for using indicators is the establishment of thresholds or another metric, often specific to each location [64]. However, as sustainability has a rather qualitative notion in policy approaches [64], it is often difficult to define a true quantitative threshold for every case [64,71].

In addition to the challenges above, the so-called “Goodhart's law” applies [78], stating that there is a high risk of missing pre-set targets by sticking too much to single indicators.

To conclude, the need and the usefulness of suitable indicators especially regarding set-up targets and monitoring the efficacy of measures is incontestable. However, even the most sophisticated indicators can cover only selected aspects of complex systems. Consequently, research on sustainable management of environmental and agronomical systems has to go beyond definition and application of selected indicators. The current age of big data paves the way for more sophisticated approaches.

### 3.4 Role of models

To estimate changes for the range of identified indicators, models can be very helpful [74,79]. To manage complex environmental and agronomic systems, existing knowledge from numerous studies is compiled, integrated, and operationalised via the development and application of the respective models [80,81]. Agroecosystem models are based on mathematical descriptions of dynamic phenomena or processes (chemical, biological, physical, etc.).

Despite the significance of empirical studies, models are considered a powerful means to up- or downscaling of results across temporal and spatial scales, for scenario analysis and risk assessment (e.g., Refs. [[82], [83], [84]]), for integration of knowledge from different scientific disciplines or simulation of interactions in complex systems [85]. In practice, however, the potential of models is often not only limited due to insufficient or uncertain input data or model parameter values but also to uncertainties related to model structure [86]. Models are based on a theory connecting the current and future/alternative system states, but this theory is often incomplete [87]. Additionally, their development requires balancing between the “degree of precision, generality and realism” [87,88]. Uncertainties of model prediction also result from global changes and relevant ecological processes (including extreme events), legacy effects, the possibility of threshold dynamics, and socio-economic impacts [62,87].

Depending on available data, purpose, and modellers’ ambitions, a variety of models of different degrees of complexity exists, partly yielding very different results when applied to the same data (e.g., Refs. [54,89]). The ability to simulate a specific system in detail with complex models comes with additional constraints, such as the number of parameters and functions compared to simple process-based models [87]. Complexity may be addressed using multi-model inference, which, however, also requires considerable resources [87]. Despite the existing idea that higher-level models can be represented through multiple instances of smaller-area models [90], this vision is simplified and bears a risk of missing cross-scale effects. Additionally, availability and reliability of models vary considerably between disciplines. Whereas models covering physical processes in environmental systems are very abundant and successful, this is much less the case in terms of chemical or biological processes, and there is no clear tendency of increasing integration of these processes into environmental system models [81,91]. E.g., existing models for ecosystem biogeochemistry are applicable mainly to homogeneous systems [51]. This is at least partly related to a fundamental problem (addressed also in subchapter 3.2 - Mono-causality): contrary to single cause-effect relationships, there is hardly any clear empirical evidence for complex interactions between various effects. Different processes need to be balanced and interlinked in complex models in arbitrary ways which have only scarcely been subjected to rigorous tests. For example, numerous models have been set up and run to study the effects of land use on hydrological processes (cf., [[92], [93], [94]]), while Lischeid et al. [95] found only minor effects of differences between various land use classes on groundwater head dynamics in a 20,000 km^2^ region. This clearly contradicts the usual expectations and demonstrates the complexity of real-world ecosystems and the importance of empirical testing and verification, rather than relying solely on models.

### 3.5 Need for empirical knowledge through tailored experiments

Intensive field experiments have been conducted till now, which provide insight into the effects of various individual factors (e.g., fertilization [96], interrelation of irrigation, tillage and crop rotation [97], topography [98], land use in watershed [99]). Yet, there is a fundamental shortage of empirical data on interactions in complex real-world processes and multi-variable relationships within the same site. That shortcoming cannot be overcome by complex models alone [62,91]. In the current era of “big data” both comprehensive data sets and powerful artificial intelligence approaches are available to fill that gap [100,101]. In fact, these approaches are now increasingly used by scientists and have been very successful in elucidating complex settings. This potential is still far from being exploited. But it has its limitations as well. A fundamental problem is the so-called “curse of dimensionality”. Data requirements of any statistical approach increase exponentially with the number of studied observables. That phenomenon is well known from experimental design theory as well as from linear statistics. It applies all the more for powerful non-linear artificial intelligence approaches (e.g., Ref. [102]), imposing substantial constraints on the design of big data studies. Making efficient use of artificial intelligence approaches requires smart experimentation designs. To that end, we propose a new generation of tailored multi-factorial experiments, that is, *landscape experiments (LE)* (please see the next chapter for explanation).

Concluding from the issues mentioned above, an important requirement of landscape experimentation in agroecosystems is that it accounts for multifaceted aspects of landscapes and the interests of various stakeholders who should be involved in defining the metrics and experimental design [30,103]. Such experiments can serve both to provide in-situ validation of simulation results or controlled experiments or for demonstration or to get insights on their own and formulate questions. They should be able to provide an opportunity to define locally relevant indicators and better reflect trade-offs across multiple dimensions [30,104]. To enable a systemic evaluation of the agroecosystem state, experimental design should account for the diversity of its interconnected functions rather than for a specific area, and the diversity of temporal and spatial scales of the respective processes. Additionally, recognition of legacy effects, as well as ongoing and expected changes (such as global changes), is important in further analysis to allow practically useful conclusions. While some initiatives have tried to address some of the issues above, we are not aware of existing examples, which fully satisfy the mentioned requirement to LE. For example, one of the most extensive examples, the USDA-ARS Experimental Watershed Network (see e.g., Ref. [105]), covers multiple sites across the USA and has a long history of observation. It is, however, rather focused on long-term monitoring activities rather than intricate interplay among various dimensions within particular agroecosystems.

## 4 Landscape experimentation

### 4.1 Differentiation of landscape experiments from “classical” field experiments

Experimentation is the foundation for successful research in the agricultural science community. We classify three major types of existing field experimentation approaches and contrast them with a new approach termed landscape experiments based on a compilation of characteristics from literature (Table 1). We define LE as a specialized research approach which is explicitly designed to investigate and gather empirical evidence on multiple processes and mechanisms that occur within an agricultural landscape (as it is defined in Chapter 2. - Current state and the need for broadening the knowledge base). These experiments utilize a fractal design, incorporating multiple interacting spatial and functional units, to systematically study natural and human-driven factors across various complexity levels. The focus of LE is on understanding the interplay of these factors at larger spatial scales and over extended periods of time, specifically relating to agroecosystems. LE challenge traditional experimental design and data analysis methods as they aim to observe the complex interactions occurring at different temporal and spatial scales simultaneously.

**Table 1:** Categorisation and key characteristics of experimental types in agricultural research. The list of publications cited is provided separately in S1.

Theme	Item	Classical field trials (FT)	Ref.	Long-term experiments (LTE)	Ref.	On-farm experiments (OFE)	Ref.	Landscape experiments (LE)	Ref.
***Physical structure***	**Size**	Small plots (1–50 m^2^)	(1)	Large plots (20 m^2^- 0.5 ha)	(2–5)	Field scale with medium plot size (200 m^2^- >10 ha in precision ag trials)	(6–8)	Landscape scale (>5 km^2^) with big plot size (>0.5 ha) or window (>1 km^2^)	(9,10, 73)
						Adequate number of spatial replicates on many farms needed	(11)	Regional scale as landscape laboratory or observatory	(12)
	**Duration**	Short-term (2–5 years)	(13)	> 20 years running time	(14)	Short-term (<1 year)	(15)	Short-term (1–5 years, focus on sampling, measurements and observations, sometimes interviews)	(16–18)
				Long-term dedication & sustained commitment	(2)	Long-term (>10 years)	(19,20)	Long-term (>10 years, focus on monitoring)	(21)
	**Treatments**	Fixed factors (e.g. variety, fertilizer rate) - ceteris paribus	(22)	Semi-fixed factors with innovation growth (varieties, machinery, fertilizer)	(23)	Fixed and contrasting factors		Manipulated landscape patterns (composition and configuration)	(24)
		Few random effects (e.g. weather, block)		Cumulative effects (e.g. SOC, total N)	(25)	Random effects over farms/locations		Multiple components & integrated topics under investigation	
		Controlled environment		More random effects (e.g. plots, block, year)	(26)			Compare a single treatment with multiple controls	(27)
	**Data collection**	Samplings and measurements		Survey samplings, repeated measurements and direct observations		Sampling without geo-referenced data	(15)	Sensing methods for landscape monitoring	(28,29)
		Replicated measurements through increase of observational units (subsamples)	(1,30,31)	Sample archive for reanalysis	(32,33)	Sampling of georeferenced data	(34)	Samplings of regular instantaneous ‘snapshots’ of entire regions over long periods (e.g. water quality)	(35)
				Unification of methodological approaches and procedures	(36)	External or commercial data acquisition (e.g. yield maps)		Grid sampling and measurements in the landscape (e.g. using chambers)	(16,29)
				Modelling (e.g. C and N cycling)	(37)	Grid sampling at field scale		Modelling (e.g. for places where direct measurement is hard)	(38,39)
								High resolution and big data (e.g. eddy-covariance)	(40)
	**Design**	Randomization, replication (mostly in space) and blocking	(41)	Randomization, replication (in time & space) and blocking	(2)	Replication preferably in space (many sites) but not in time	(7)	Pseudoreplicates as true replicate of landscapes is seldom possible	(24,35,42)
		Factorial design	(43)	Inclusion of control (standard treatment) plots (Business as usual)		Farmer business as usual as main comparison		Complex interactions and interference	(10)
		as Randomized complete block design, Latin Square or Alpha-lattice	(22)	Non-uniform correlation structure of within-plot observations	(44)	Simple factor comparison, reduction of interactions		Laid out under real-life conditions, randomization limited but representativeness maximized	(29,45)
				with Fixed or staggered start (for crop rotations)	(46)	as Strips, whole-of block or split fields, contoured plots or (randomized) chequerboards	(11,34,47)	by regular/random/transect/stratified/cluster/contour sampling (depending on homogeneity of included measured landscape properties)	(46, 47)
***Function***	**Statistical properties**	Simple and well documented		Advanced and well documented		Ongoing and well-documented		Complex and unsolved	
		Variance and mean (ANOVA)		Mixed models and REML estimation	(26)	Local/global estimation	(15)	General linear mixed models	(50)
		Regression model		Time series analysis and temporal autocorrelation		Anisotropic models	(51)	Hierarchical Bayesian models	
				Repeated measures and spatio-temporal autocorrelation	(3)	Multivariate geostatistics	(52)	Time series, Repeated measures and autocorrelation	(28,53,54)
				Mixed model analysis by residual (restricted) maximum likelihood estimation,	(44)			PCA	(49, 74)
				ANOVA & ANCOVA	(55)			Multiple regression analysis, PERMANOVA	(56,57, 75, 76, 77)
								(Co)-Kriging, State-space analysis, Spectral analysis	
	**Research target**	Genotyping for suitability to local conditions, high yields		Cropping system comparison		Farmer-based approach to experimentation		Change in trends & extension of results at regional scale	
		Efficacy of fertilization (4R)		Soil fertility and nutrient cycling	(58)	Productivity, profitability	(47)	Spatial and temporal variation in responses of crops and soils to natural and anthropogenic factors	(29,56)
		Crop protection against pests, diseases and weeds		Yield stability	(3)	Enabling digital agriculture	(47)	Identification and quantification of key landscape elemental features, fluxes and processes	(59–61)
						Capture and utilize spatial and temporal variability	(47)		
	**Main focus**	Studying isolated components	(13)	Sustainability, environmental quality, adaptation to change	(62)	Alternative (improved) management		Ecology and biodiversity	(50,63)
		Cultivar evaluation		Identification of long-term trends	(58)	Precision agriculture (Site-specific crop management treatment)		Descriptive agroecosystem studies	
		Test of agronomic management practices		Understanding the past and present ecosystem dynamics	(64)			Agroecology and sustainable agricultural practices	(65,66)
	**Specific challenges**	Soil homogeneity	(67)	Inherent soil variability increases residual error	(68)	Machinery requirements		Complexity of increasing scale and duration, funding	(35,65)
		Edge effects	(69)	Long-term funding	(58)	Stakeholder involvement	(70)	Statistical analysis, modelling and scaling	(24)
		Interplot competition	(71,72)			Policy linkage		Large potential for unaccounted confounding effects	(27)

In contrast, typical controlled and randomized field experiments comprise classical field trials (FT) and long-term experiments (LTE) varying in their execution time. Meanwhile, on-farm experiments (OFE) are set up in less clearly controlled and heterogeneous environments. FT and LTE studies aim at keeping soil heterogeneity and climate variability as small as possible during the study period whereas OFE are usually characterized by pragmatic compromises between the desirable and the feasible variability. In contrast, LE consider heterogeneity as a source of information that needs to be addressed explicitly. Traditional experimentation under controlled conditions is carried out to study a single effect or process under ceteris paribus conditions which increases the mechanistic understanding but ignores more complex interactions with the surroundings by design. This in turn is raised especially in LE, whereas OFE are carried out frequently under heterogeneous soil conditions, though trying to control the field variability with large plots, random treatment allocation and blocking. The large plots also justify the limited number of treatments commonly investigated in OFE and explain the magnitude of high spatial resolution data from yield maps and sensing. Contrarily to OFE, FT and LTE provide a vast amount of systematically manually collected data and covariates to answer research questions and confirm or reject hypotheses. While OFE and FT may ignore relevant interactions and trends in their short implementation period, LTE and LE include a wider range of spatial or temporal entities to support a better understanding of interactions and interdependence. While FT, LTE and OFE often request for randomized allocation of treatments in space, LE intentionally select the most representative and accessible experimental site for the processes under observation, considering both, transition zones and boundary effects. There exists a sharp gradient in the involvement of external stakeholders in the design and development of experiments, encouraging many external stakeholders (mostly farmers, industry and regional authorities) in OFE, sporadically in LE and exceptionally in LTE (broader involvement of stakeholders e.g., NGOs, ministries and industry), but excluding them in FT. Other features, barriers and opportunities for landscape experimentation are provided in the subsequent chapters and Table 1.

The combination of different experimental types is possible and sometimes mandatory to answer particular research questions. Experimental results of controlled on-station and farmer-centric on-farm trials can be combined to provide a balanced evaluation for farmers′ practical feasibility and scientific assessment [15]. OFE can be designed and carried out in the long-term (“Long-term on-farm experiment”) to monitor changes in yield and soil under real-world conditions [106]. In many large-scale LE, smaller plots and FT have been installed to focus on particular process dynamics [107,108]. Other LEs are implemented under on-farm conditions in small experimental plots within a larger radius of a surrounding area under monitoring [109]. There are several examples available for short-term, small-scale and highly instrumented experiments in landscape research involving long-term monitoring activities that belong to networks like the Integrated European Long-Term Ecosystem Research (eLTER [110]) or International Long-Term Ecological Research (ILTER [111]).

### 4.2 Implications for experimental design

Through an extensive examination of the available literature our review unrevealed a lack of sound approaches for the experimental design of LE. The requirements of LE exceed those of classical field experiments by far. First, while LE aim at elucidating the complex interplay between numerous effects, a direct consequence is the curse of dimensionality in terms of required treatments. Thus, a full factorial design is far from feasible. In addition, a ceteris paribus design cannot be realised. Even more, real replicates can hardly be implemented in a high-dimensional experimental setup. I.e., a change in the landscape cannot be repeated or controlled and thus cannot be easily compared as it can be done in designed factorial treatment experiments. At the same time, the usual postulate of field trials being representative for a larger region can and has to be dropped for high-dimensional settings.

LEs are, however, characterized by uncertain definitions of spatial and temporal entities in the agricultural science context (see also Pereponova et al., 2023, in review). As the focus of LE is on interactions, the surroundings of the experiments are integral parts of the experiment and should be covered by the measurements as well. I.e., farms are embedded within larger ecological and human systems with a large diversity of biophysical and cultural components. Such interactions take place within and between different entities and may include macro-scale feedbacks or cross-scale interactions or emergence, potentially leading to novel or unexpected system behaviour. Data at both farm- and landscape levels are needed that depict the diversity of conditions, accounting both for within- and between-farm heterogeneity, including biophysical (usually public), economic and site-specific (often confidential) data [62]. Sampling design may play an important role in representing the heterogeneity accounting for the large complexity of interactions. Patterns, caused by various processes at different spatial scales, may be detected using a combination of the sampling design - e.g., by analysing transect data of study sites (see e.g., Ref. [112]) - and other analytical approaches.

There is also a need for site-specific data. In addition, boundary effects need to be accounted for rather than assuming homogeneity or purely random heterogeneity within the single treatments. Thus, in contrast to classical field trials, location matters both within and beyond the treatments. Consequently, data collection has to be performed at high spatial and temporal resolution. Boundary conditions are hardly constant or reproducible which implies that temporal replicates can hardly be implemented, but there is a need to capture the dynamics. Landscape (as defined in Chapter 2), can serve as a suitable scale for the purposes of research on agroecology. Shaped by relationships between its elements, rather than being defined by a particular size, it supports the idea that (agro)ecosystems should be considered as dynamic and “interacting, self-organized sets of processes and structures”, varying across scales [46]. It allows reflecting on the most important changes, including the effects of political or individual decisions and of ecological processes occurring at different scales [11,31,65,113,114].

For better understanding of the impact of future climate-related changes on agricultural systems, possible time lags and effects of “slow variables” [115] on the system, long-term observations in landscape experiments should be on the agenda.

An inevitable consequence of this rather ambitious concept is that LE require unprecedented methods for data acquisition, as compared to classical field trials. Meanwhile, sensors and data are now available at fairly cheap costs and effort. New technologies allow automated data collection at a large scale (such as UAV or other remote sensing techniques for the observation of soil parameters or plant health in relation to e.g., pest, disease or weed pressure). Additionally, LE can be used in combination with smaller field trials to investigate more specific questions. Fig. 1 provides examples of research topics within the corresponding scales and the types of experiments typically used to address these topics, as well as the different data collection methods by the scales they cover. Further, LE are foreseen to be implemented as a part of larger transdisciplinary projects (such as Living Labs). While Landscape Experiments (LE) incorporate stakeholder perspectives and an understanding of demands and potential limitations through co-design elements, they aim to provide evidence of success of tested measures or practices from an economic and agri-environmental perspective. Altogether, this leads to better transferability of the results.

Since LE requires continuous cooperation of interdisciplinary colleagues and practitioners, standardized experimental design is critical with thorough and harmonized documentation and sharing of collected data (e.g., via well-aligned data management plans and data repositories). Such standards still need to be developed and tested.

### 4.3 Implications for data analysis

A second challenge that our review identified is the need for more complex statistical and advanced data analysis methods to grasp the complexity of LE. From the requirements above it is clear that common textbook knowledge on experimental design cannot be applied, since e.g., there is no control to compare treatment effects with, neither in space nor in time. Additionally, it would not suffice to test effects for significance only, but effect size needs to be quantified for the respective boundary conditions to allow comparison with other effects. Sound foundations for the analysis of landscape experiments exist only in single preliminary fragments. But a first outline is given below.

A major challenge is a quantitative delineation of different effects in complex settings. Here, existing dimensionality reduction approaches are the first choice, like Non-Metric Dimensional Scaling, Principal Component Analysis (PCA; e.g. Ref. [116]), Empirical Orthogonal Functions [117], Isometric Feature Mapping [118], Kernel PCA [119], etc. In addition, modern machine learning approaches are very versatile and powerful tools in that regard. Rillig et al. [58] gave a nice example of how to use machine learning to study the interplay between various effects in a complex multi-factorial experiment. In addition, powerful visualisation tools like Self-Organising Maps combined with Sammon Mapping help to structure the analysis (e.g., Ref. [120]). Structural Equation Modelling (e.g., Refs. [121,122]) is widely used to study complex relationships but is usually restricted to linear dependencies.

Two of the Nobel Prize winners in economics in 2021 draw attention to a sophisticated toolbox to study causal relationships in complex settings far from clearly defined experiments. The toolbox comprises the matching approach [123], assessment of instrumental variables [124] and regression discontinuity design [125]. This work is largely unknown in agronomy and landscape research but obviously has great potential for the analysis of the outcome of landscape experiments. Last but not least, mechanistic models can be used as well. However, these models need to be designed specifically for hypothesis testing which is rarely the case (e.g., Ref. [126]).

Antle et al. [62] highlighted the need for a system approach in new-generation models to assess the sustainability of agricultural systems, where different components could be potentially represented by modular sub-systems. However, their integration is not straightforward, and it is important to recognize the level, to which this modularization is possible. Standards for linking between different subsystem elements are needed. Additionally, uncertainty about how an ecological process will interact with changing conditions still applies, e.g., if the experiment is intended to inform decisions on proactive management measures, or when spatial scaling is applied. Nevertheless, hybrid models which combine ecological mechanisms and correlational components and link statistically extrapolated changes to processes may be explored [87].

Among recent developments, Digital Twins are believed to offer an opportunity to enable better process understanding, which utilize real-world data, simulation, or machine learning models combined with data analysis. For the moment, however, the research on agricultural Digital Twins remains limited [127].

**Fig. 2:**
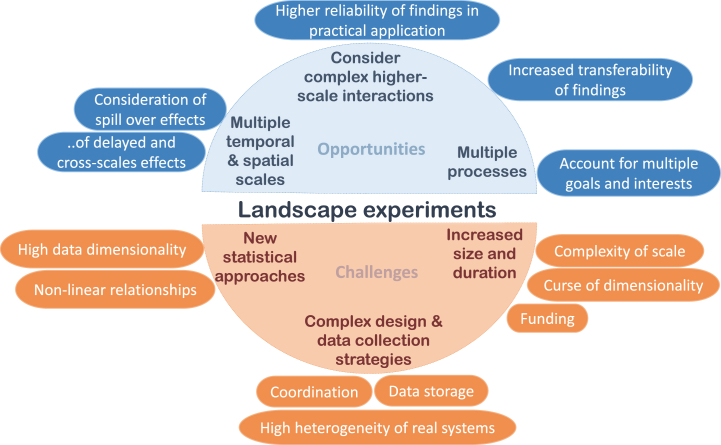
Key opportunities and challenges in landscape experiments.

We summarize the opportunities and challenges of LE in Fig. 2.

### 4.4 AgroScapeLab quillow and patchCROP as examples of landscape experiments

Knowing about the lack of research in agricultural landscape settings, institutional solutions for landscape experimentation outline pathways to integrate large plot sizes, long running time, high-resolution data from different disciplines, and innovate data management and analysis to comply with multiple processes and mechanisms that occur within an agricultural landscape. This section will present two examples of existing landscape experiments and highlight their key features that make them particularly representative as LE examples.

First, in the case of the AgroScapeLab Quillow (ASLQ, www.aslq.de), a large-scale agricultural landscape observatory was set up in 2011 in a 170 km^2^ catchment area in Northern Brandenburg. The ASLQ comprises three key components of LE: (i) landscape manipulation experiments that extend classical FT to larger scales and often involve farmers through OFE, (ii) a technology platform for developing and testing emerging technologies of precision agriculture (e.g., tractor-based sensor networks, soil sensing, etc.), and (iii) long-term monitoring to detect spatio-temporal trends, slow shifts or delayed response of landscape processes. Integrated research carried out in the ASLQ aims to develop a genuine landscape research approach by merging comprehensive data sets from different disciplines including geosciences, hydrology, microbiology, ecology, soil science, and agronomy and using modern data mining and systems analysis approaches. This way, a systematic understanding of numerous interdependencies and feedback loops at the landscape scale can be reached and knowledge transfer in agriculture can be addressed.

Second, in the case of patchCROP (www.landschaftslabor-patchcrop.de), a LE case study was launched in March 2020 covering about 400 ha of an agricultural landscape of a single farm in Eastern Brandenburg. In patchCROP, a multidisciplinary research approach is applied to investigate how diversified agricultural landscapes of the future can be created through small-scale and site-adapted cultivation [128]. These novel cropping systems are being developed and scaled using digitization and new technologies such as robotics, sensors and artificial intelligence. A targeted, multidisciplinary and comprehensive measurement and monitoring plan was developed together with a project-specific data management plan, which bindingly regulates for all project partners which data were and are collected when, where and how. Maintaining this data management plan, however, poses several challenges such as integration and storage of large amounts of data, data sharing and providing access, data security and documentation, as well as coordination within a large team of collaborators.

Experimental infrastructures like the ASLQ or patchCROP address innovative agricultural research experimentation for sustainable crop production from different points of view: firstly, the farmer-centric position focusing on the economic return and feasibility of future field operations, secondly, the scientific perspective evaluating dynamic, interdependent or opposing processes like changes in biodiversity and related ecosystem services and address interests and trade-offs of and for different stakeholders. Thirdly, the technical dimension of innovative research is present for state-of-the-art data collection and analysis, as a step to address the “curse of dimensionality” problem.

However, we are aware that ensuring sufficient resources and funding throughout the LE's lifespan can be difficult, especially in the Global South. Currently, the maintenance of the basic research infrastructures ASLQ and patchCROP is provided by institutional household funding, whereas additional investments of devices, technologies and costly variables are financed through third-party funding and docking projects. To guarantee enriching synergies and exchange of data and knowledge between disciplines, a LE needs to be handled with platform character, keeping it open to attract other research institutions as well. This solution is also feasible outside the Global North to ensure multidisciplinary data collection and can be initiated to open doors for institutional LTE or OFE.

Other barriers for LE are related to the high complexity of coordination efforts and collaboration within a larger group of partners, and higher requirements for data storage and sharing.

## 5 Conclusions

To provide a scientific base for policies and management that support the transformation of agriculture, research should account for the diverse interests of stakeholders and relevant goals, including environmental, economic or social aspects simultaneously, as is the core of agroecology in its current understanding. The agroecosystem level of agroecology is the first on the agenda, which should account for the processes within and between the productive fields and their surroundings. Existing methods are limited in this regard. Specifically, we identified shortcomings of existing studies such as focus on single scales to address selected processes and primarily enabling the testing of predefined hypotheses. This carries a risk of disregarding unexpected cause-effect relationships within or across spatial and temporal scales and is closely related to the second challenge, which is a limited ability to account for multiple factors and their relationships. These shortcomings stem from the limitations of evidence acquisition (mainly experimentation) and data analysis methods currently employed in agricultural research. To address these problems, we suggest that a next generation of landscape experimental approaches needs to be established that accounts for these drawbacks of traditional experimentation methods. Though the approaches regarding study design and data analysis are still incomplete to meet the ambitious goals and requirements for LE, the first outline and ideas on further development are provided in this article, illustrated by existing examples. Alongside methodological constraints, a practical requirement is that funding programmes and instruments are in place to support this type of research and ensure long-term and spatially extensive incentives for conducting landscape experimentation.

## CRediT authorship contribution statement

**Anna Pereponova:** Conceptualization, Formal analysis, Visualization, Writing – original draft, Writing – review & editing. **Kathrin Grahmann:** Conceptualization, Formal analysis, Visualization, Writing – original draft, Writing – review & editing. **Gunnar Lischeid:** Conceptualization, Formal analysis, Writing – original draft, Writing – review & editing. **Sonoko Dorothea Bellingrath-Kimura:** Writing – review & editing. **Frank A. Ewert:** Writing – review & editing.

## Declaration of competing interest

The authors declare that they have no known competing financial interests or personal relationships that could have appeared to influence the work reported in this paper.
