# Supplementary material for: Sustainable transformation of agriculture requires landscape experiments
Source: Heliyon. 2023 Oct 24;9(11):e21215. doi: 10.1016/j.heliyon.2023.e21215 (PMC10641153; doi:10.1016/j.heliyon.2023.e21215)
Supplement: Multimedia component 1 [file mmc1.docx]

**Sustainable transformation of agriculture requires landscape experiments**

**List of the literature used for Table 1**

1. Casler MD. Fundamentals of experimental design: Guidelines for designing successful experiments. Agronomy Journal. 2015;107(2):692–705.

2. McRae KB, Ryan DAJ. Design and planning of long-term experiments. Canadian Journal of Plant Science. 1996;76(4):595–602.

3. Reckling M, Ahrends H, Chen TW, Eugster W, Hadasch S, Knapp S, et al. Methods of yield stability analysis in long-term field experiments. A review. Agronomy for Sustainable Development. 2021;41(2).

4. Thai TH, Omari RA, Barkusky D, Bellingrath-Kimura SD. Statistical analysis versus the m5p machine learning algorithm to analyze the yield of winter wheat in a long-term fertilizer experiment. Agronomy. 2020;10(11):1–19.

5. Grahmann K, Rubio Dellepiane V, Terra JA, Quincke JA. Long-term observations in contrasting crop-pasture rotations over half a century: Statistical analysis of chemical soil properties and implications for soil sampling frequency. Agriculture, Ecosystems & Environment. 2020 Jan;287:106710.

6. Lightfoot C, Barker R. On-farm trials: A survey of methods. Agricultural Administration and Extension. 1988;30(1):15–23.

7. Fielding WJ, Riley J. Aspects of design of on-farm fertilizer trials. Experimental Agriculture. 1998;34(2):219–30.

8. Trevisan RG, Bullock DS, Martin NF. Spatial variability of crop responses to agronomic inputs in on-farm precision experimentation. Precision Agriculture. 2021;22(2):342–63.

9. Atangana A, Khasa D, Chang S, Degrande A. Tropical agroforestry. Tropical Agroforestry. 2014;1–380.

10. Cunningham RB, Lindenmayer DB. Approaches to Landscape Scale Inference and Study Design. Current Landscape Ecology Reports. 2017;2(1):42–50.

11. Yan W, Hunt LA, Johnson P, Stewart G, Lu X. On-farm strip trials vs. replicated performance trials for cultivar evaluation. Crop Science. 2002;42(2):385–92.

12. Toffolini Q, Capitaine M, Hannachi M, Cerf M. Implementing agricultural living labs that renew actors’ roles within existing innovation systems: A case study in France. Journal of Rural Studies. 2021;88(October):157–68.

13. Drinkwater LE. Cropping systems research: Reconsidering agricultural experimental approaches. HortTechnology. 2002;12(3):355–61.

14. Grosse M, Hierold W, Ahlborn MC, Piepho HP, Helming K. Long-term field experiments in Germany: Classification and spatial representation. Soil. 2020;6(2):579–96.

15. Piepho HP, Richter C, Spilke J, Hartung K, Kunick A, Thöle H. Statistical aspects of on-farm experimentation. Crop and Pasture Science. 2011;62(9):721–35.

16. Redlich S, Zhang J, Benjamin C, Dhillon MS, Englmeier J, Ewald J, et al. Disentangling effects of climate and land use on biodiversity and ecosystem services—A multi-scale experimental design. Methods in Ecology and Evolution. 2022;13(2):514–27.

17. Hou L, Liu P, Huang J, Deng X. The influence of risk preferences, knowledge, land consolidation, and landscape diversification on pesticide use. Agricultural Economics (United Kingdom). 2020;51(5):759–76.

18. Hass AL, Kormann UG, Tscharntke T, Clough Y, Baillod AB, Sirami C, et al. Landscape configurational heterogeneity by small-scale agriculture, not crop diversity, maintains pollinators and plant reproduction in western Europe. Proceedings of the Royal Society B: Biological Sciences. 2018;285(1872).

19. Schirrmann M, Joschko M, Gebbers R, Kramer E, Zörner M, Barkusky D, et al. Proximal soil sensing - A contribution for species habitat distribution modelling of earthworms in agricultural soils? PLoS ONE. 2016;11(6):1–21.

20. de Oliveira Ferreira A, Amado TJC, Rice CW, Gonçalves DRP, Ruiz Diaz DA. Comparing on-farm and long-term research experiments on soil carbon recovery by conservation agriculture in Southern Brazil. Land Degradation and Development. 2021;32(11):3365–76.

21. Lindenmayer DB, Likens GE, Andersen A, Bowman D, Bull CM, Burns E, et al. Value of long-term ecological studies. Austral Ecology. 2012;37(7):745–57.

22. Schmidt P, Möhring J, Koch RJ, Piepho HP. More, larger, simpler: How comparable are on-farm and on-station trials for cultivar evaluation? Crop Science. 2018;58(4):1508–18.

23. Frye WW, Thomas GW. Management of Long-Term Field Experiments. Agronomy Journal. 1991;83(1):38–44.

24. Jenerette GD, Shen W. Experimental landscape ecology. Landscape Ecology. 2012;27(9):1237–48.

25. Franzluebbers AJ, Stuedemann JA. Soil-profile distribution of organic C and N after 6years of tillage and grazing management. European Journal of Soil Science. 2013;64(5):558–66.

26. Onofri A, Seddaiu G, Piepho HP. Long-Term Experiments with cropping systems: Case studies on data analysis. European Journal of Agronomy. 2016;77:223–35.

27. Oksanen L. Logic of experiments in ecology: Is pseudoreplication a pseudoissue? Oikos. 2001;94(1):27–38.

28. Webster R, Payne RW. Analysing repeated measurements in soil monitoring and experimentation. European Journal of Soil Science. 2002;53(1):1–13.

29. Mueller L, Sychev VG, Dronin NM, Eulenstein F. Exploring and Optimizing Agricultural Landscapes. Mueller L, Sychev VG, Dronin NM, Eulenstein F, editors. Innovations in Landscape Research Exploring and Optimizing Agricultural Landscapes. Cham: Springer International Publishing; 2021. 679–691 p. (Innovations in Landscape Research).

30. Perrett JJ. A method for analyzing unreplicated experiments using information on the intraclass correlation coefficient. Journal of Modern Applied Statistical Methods. 2006;5(2):432–42.

31. Perrett JJ, Higgins JJ. A method for analyzing unreplicated agricultural experiments. Crop Science. 2006;46(6):2482–5.

32. Richter D deB. B, Hofmockel M, Callaham MA, Powlson DS, Smith P. Long-term soil experiments: Keys to managing Earth’s rapidly chancing ecosystems. Soil Science Society of America Journal. 2007;71(2):266–79.

33. Ayres E. Quantitative Guidelines for Establishing and Operating Soil Archives. Soil Science Society of America Journal. 2019;83(4):973–81.

34. Panten K, Bramley RGV, Lark RM, Bishop TFA. Enhancing the value of field experimentation through whole-of-block designs. Precision Agriculture. 2010;11(2):198–213.

35. Hargrove WW, Pickering J. Pseudoreplication: a sine qua non for regional ecology. Landscape Ecology. 1992;6(4):251–8.

36. Grahmann K, Terra JA, Ellerbrock R, Rubio V, Barro R, Caamaño A, et al. Data accuracy and method validation of chemical soil properties in long-term experiments: Standard operating procedures for a non-certified soil laboratory in Latin America. Geoderma Regional. 2022;28.

37. Baethgen WE, Parton WJ, Rubio V, Kelly RH, M. Lutz S. Ecosystem dynamics of crop–pasture rotations in a fifty-year field experiment in southern South America: Century model and field results. Soil Science Society of America Journal. 2021;85(2):423–37.

38. Sun W, Liu X. Review on carbon storage estimation of forest ecosystem and applications in China. Vol. 7, Forest Ecosystems. SpringerOpen; 2020.

39. Hernández-Ochoa IM, Gaiser T, Kersebaum KC, Webber H, Seidel S, Grahmann K, et al. Model-based design of crop diversification through new field arrangements in spatially heterogeneous landscapes. A review. Agronomy for Sustainable Development. 2022;x:1–47.

40. Freitag U, Fuchs-Kittowski F, Abecker A, Hosenfeld F. Umweltinformations- systeme – Wie verändert die Digitalisierung unsere Gesellschaft? In: Tagungsband des 27 Workshops des Arbeitskreises „Umweltinformationssysteme“ der Fachgruppe „Informatik im Umweltschutz‘‘ der Gesellschaft für Informatik (GI) 2020. Nomos Verlagsgesellschaft mbH & Co. KG; 2020. p. 53–65.

41. Fisher R. The Design of Experiments. 4th ed. Edinburgh : Oliver and Boyd; 1947.

42. Davies GM, Gray A. Don’t let spurious accusations of pseudoreplication limit our ability to learn from natural experiments (and other messy kinds of ecological monitoring). Ecology and Evolution. 2015;5(22):5295–304.

43. Montgomery DCASU. D esign and Analysis of Experiments Ninth Edition. 2017. 640 p.

44. Payne RW. The design and analysis of long-term rotation experiments. Agronomy Journal. 2015;107(2):772–85.

45. McPhee C, Bancerz M, Mambrini-Doudet M, Chrétien F, Huyghe C, Gracia-Garza J. The defining characteristics of agroecosystem living labs. Sustainability (Switzerland). 2021;13(4):1–25.

46. Loughin TM. Improved experimental design and analysis for long-term experiments. Crop Science. 2006;46(6):2492–502.

47. Lacoste M, Cook S, McNee M, Gale D, Ingram J, Bellon-Maurel V, et al. On-Farm Experimentation to transform global agriculture. Nature Food. 2021;

48. FAO. Measuring and modelling soil carbon stocks and stock changes in livestock production systems. 2018.

49. Bowles TM, Acosta-Martínez V, Calderón F, Jackson LE. Soil enzyme activities, microbial communities, and carbon and nitrogen availability in organic agroecosystems across an intensively-managed agricultural landscape. Soil Biology and Biochemistry. 2014 Jan;68:252–62.

50. Geiger F, Bengtsson J, Berendse F, Weisser WW, Emmerson M, Morales MB, et al. Persistent negative effects of pesticides on biodiversity and biological control potential on European farmland. Basic and Applied Ecology. 2010 Mar;11(2):97–105.

51. Tanaka TST. Assessment of design and analysis frameworks for on-farm experimentation through a simulation study of wheat yield in Japan. Precision Agriculture. 2021;

52. Jin H, Shuvo Bakar K, Henderson BL, Bramley RGV, Gobbett DL. An efficient geostatistical analysis tool for on-farm experiments targeted at localised treatment. Biosystems Engineering. 2021;205:121–36.

53. Finney DJ. Repeated measurements: What is measured and what repeats? Statistics in Medicine. 1990;9(6):639–44.

54. Wauchope HS, Amano T, Geldmann J, Johnston A, Simmons BI, Sutherland WJ, et al. Evaluating Impact Using Time-Series Data. Trends in Ecology and Evolution. 2021;36(3):196–205.

55. Heil K, Schmidhalter U. Improved evaluation of field experiments by accounting for inherent soil variability. European Journal of Agronomy. 2017;89(November 2016):1–15.

56. Veldkamp A, Kok K, De Koning GHJ, Schoorl JM, Sonneveld MPW, Verburg PH. Multi-scale system approaches in agronomic research at the landscape level. Soil and Tillage Research. 2001 Mar;58(3–4):129–40.

57. Anderson MJ. Permutational Multivariate Analysis of Variance ( PERMANOVA ) . Wiley StatsRef: Statistics Reference Online. 2017;1–15.

58. Berti A, Marta AD, Mazzoncini M, Tei F. An overview on long-term agro-ecosystem experiments: Present situation and future potential. European Journal of Agronomy. 2016;77:236–41.

59. Butterbach-Bahl K, Baggs EM, Dannenmann M, Kiese R, Zechmeister-Boltenstern S. Nitrous oxide emissions from soils: how well do we understand the processes and their controls? Philosophical Transactions of the Royal Society B: Biological Sciences. 2013 Jul 5;368(1621):20130122.

60. Schmugge TJ, Kustas WP, Ritchie JC, Jackson TJ, Rango A. Remote sensing in hydrology. Advances in Water Resources. 2002 Aug;25(8–12):1367–85.

61. Sun W, Liu X. Review on carbon storage estimation of forest ecosystem and applications in China. Forest Ecosystems. 2020 Dec 30;7(1):4.

62. Rasmussen PE, Goulding KWT, Brown JR, Grace PR, Janzen HH, Korschens M. Long-term agroecosystem experiments: Assessing agricultural sustainability and global change. Science. 1998;282(5390):893–6.

63. Kremen C, Williams NM, Aizen MA, Gemmill-Herren B, LeBuhn G, Minckley R, et al. Pollination and other ecosystem services produced by mobile organisms: a conceptual framework for the effects of land-use change. Ecology Letters. 2007 Apr;10(4):299–314.

64. Jandl R, Rodeghiero M, Martinez C, Cotrufo MF, Bampa F, van Wesemael B, et al. Current status, uncertainty and future needs in soil organic carbon monitoring. Science of the Total Environment. 2014;468–469:376–83.

65. Jeanneret P, Aviron S, Alignier A, Lavigne C, Helfenstein J, Herzog F, et al. Agroecology landscapes. Landscape Ecology. 2021;36(8):2235–57.

66. Clancy MA. Forest Soil Carbon Stocks and Life Cycle Assessment of Short Rotation Forestry. 2018;(November).

67. Becher HH. On the Importance of Soil Homogeneity when Evaluating Field Trials. Journal of Agronomy and Crop Science. 1995;174(1):33–40.

68. Poeplau C, Bolinder MA, Kätterer T. Towards an unbiased method for quantifying treatment effects on soil carbon in long-term experiments considering initial within-field variation. Geoderma. 2016;267:41–7.

69. Raatz L, Bacchi N, Pirhofer Walzl K, Glemnitz M, Müller MEH, Joshi J, et al. How much do we really lose?—Yield losses in the proximity of natural landscape elements in agricultural landscapes. Ecology and Evolution. 2019;9(13):7838–48.

70. Neef A, Neubert D. Stakeholder participation in agricultural research projects: A conceptual framework for reflection and decision-making. Agriculture and Human Values. 2011;28(2):179–94.

71. Rebetzke GJ, Fischer RA, Van Herwaarden AF, Bonnett DG, Chenu K, Rattey AR, et al. Plot size matters: Interference from intergenotypic competition in plant phenotyping studies. Functional Plant Biology. 2014;41(2):107–18.

72. Talbot M, Milner AD, Nutkins MAE, Law JR. Effect of interference between plots on yield performance in crop variety trials. The Journal of Agricultural Science. 1995 Jun 27;124(3):335–42.

73. Bretagnolle V, Berthet E, Gross N, Gauffre B, Plumejeaud C, Houte S et al. Towards sustainable and multifunctional agriculture in farmland landscapes: Lessons from the integrative approach of a French LTSER platform. Sci Total Environ 2018; 627:822–34.

74. Cushman SA, McGarigal K, Neel MC. Parsimony in landscape metrics: Strength, universality, and consistency. Ecological Indicators 2008; 8(5):691–703.

75. Yang Y, Jia X, Wendroth O, Liu B. Estimating Saturated Hydraulic Conductivity along a South-North Transect in the Loess Plateau of China. Soil Science Society of America Journal 2018; 82(5):1033–45.

76. Wendroth O, Reynolds WD, Vieira SR, Reichardt K, Wirth S. Chapter 11 Statistical approaches to the analysis of soil quality data. In: Soil Quality for Crop Production and Ecosystem Health. Elsevier; 1997. p. 247–76 (Developments in Soil Science).

77. Rodrigues FA, Blasch G, Defourny P, Ortiz-Monasterio JI, Schulthess U, Zarco-Tejada PJ et al. Multi-Temporal and Spectral Analysis of High-Resolution Hyperspectral Airborne Imagery for Precision Agriculture: Assessment of Wheat Grain Yield and Grain Protein Content. Remote Sens (Basel) 2018; 10(6):930.
